# Recruitment of Adolescent Girls and Young Women into an Early Oral PrEP Open- Label Study in Southern Africa: Lessons Learned from HPTN 082

**DOI:** 10.21203/rs.3.rs-5084642/v1

**Published:** 2025-03-28

**Authors:** Miliswa Magongo, Nomsa Mhlanga, Sisa Nobanda, Charles Chasakara, Ntando Yola, Kathy Hinson, Marcus Bryan, Phumeza Mzizi, Thandekile Essien, Nicholas Hastings, Makhosazane Nomhle Ndimande-Khoza, Linda-Gail Bekker, Nyaradzo Mgodi, Connie Celum, Sinead Delany-Moretlwe

**Affiliations:** Wits RHI, University of the Witwatersrand; University of Zimbabwe Clinical Trials Research Centre; Desmond Tutu Health Centre, University of Cape Town; University of Zimbabwe Clinical Trials Research Centre; Desmond Tutu Health Centre, University of Cape Town; Family Health International (FHI 360); Family Health International (FHI 360); Wits RHI, University of the Witwatersrand; Wits RHI, University of the Witwatersrand; Family Health International (FHI 360); Wits RHI, University of the Witwatersrand; Desmond Tutu Health Centre, University of Cape Town; University of Zimbabwe Clinical Trials Research Centre; University of Washington; Wits RHI, University of the Witwatersrand

**Keywords:** pre-exposure prophylaxis, AGYW, GPP, community engagement, HIV prevention, clinical trials, Sub-Saharan Africa

## Abstract

**Background:**

Adolescent girls and young women (AGYW) in southern Africa who are at high risk for HIV acquisition can mitigate this risk by using daily oral pre-exposure prophylaxis (PrEP) consistently. Using reflections from the community engagement teams in an early oral PrEP trial, this paper presents lessons learned from recruiting AGYW into the trial. It highlights experiences and strategies employed during the planning, readiness, and implementation phases of the trial.

**Methods:**

The HIV Prevention Trials Network (HPTN) 082 was an open-label study of PrEP uptake and adherence conducted between October 2016 and October 2018 among 16- to 25-year-old women without HIV in Cape Town and Johannesburg, South Africa, and Harare, Zimbabwe. A joint community team meeting with team members from all three HPTN 082 sites garnered and synthesized team experiences by analysing fieldwork reflections, HPTN 082 study-led workshop summaries, project records, and Community Advisory Board (CAB) meeting minutes about lessons for stakeholder engagement that are relevant for PrEP introduction and service delivery.

**Results:**

Using the Good Participatory Practice (GPP) framework, thighlighted the value of PrEP education, engagement with stakeholders during the formative phase, and the importance of peers and family as sources of information, support, and referral for adolescent study participants. In the first year of recruitment for HPTN 082, study participants reported they needed support for consistent daily oral PrEP use from parents and other adults, and efforts were intensified to engage parents and community stakeholders.

**Conclusions:**

The introduction of daily oral PrEP, a novel HIV prevention for young African AGYW, required multiple strategies that were culturally sensitive, age-appropriate, and included peers, partners, parents, and other adults who influence health behaviours in AGYW.

**Trial registration:**

Clinicaltrials.gov NCT02732730, 13 November 2018.

## Background

In 2015, the World Health Organization (WHO) recommended daily oral pre-exposure prophylaxis (referred to as “PrEP”) as part of a comprehensive HIV prevention package (1). Countries adopted PrEP in national guidelines for populations at risk (2–4). Given ongoing high incidence of HIV among adolescent girls and young women (AGYW) aged 15 to 25 years old in sub-Saharan Africa, they are priority populations for the offer of PrEP (5). There were concerns however about whether AGYW would use PrEP consistently, following results from earlier trials that indicated very low PrEP adherence in younger women, although HPTN 067/ADAPT **[NCT01327651]** provided reassurance that young women could adhere to daily dosing of open-label oral PrEP with adequate support (6–8).

Following the 2015 WHO recommendations, countries-initiated processes to support PrEP introduction into national programmes. In South Africa, a PrEP technical working group was established to advise on the National Policy on HIV PrEP and Test and Treat (9). Beginning mid-June 2016, oral PrEP was included as part of a package of care for sex workers, and by the end of 2016, oral PrEP had been introduced in 12 clinics in five South African provinces. This rollout did not initially specifically include AGYW (10). The inclusion of AGYW in PrEP programmes in South Africa only took place in 2018. Zimbabwe adopted the WHO global guidance on PrEP in 2015, and subsequently launched it as part of the antiretroviral therapy (ART) consolidated guidelines in December 2016 (9) but did not scale up PrEP provision until after 2017.

Ahead of national PrEP introductions, and in part to understand factors influencing PrEP use in AGYW, several oral PrEP demonstration projects were conducted to assess uptake and adherence (11, 12). The HIV Prevention Trials Network (HPTN) 082 study was one such study that aimed to demonstrate PrEP uptake and adherence among sexually active AGYW without HIV aged 16 to 25 living in Cape Town and Johannesburg, South Africa, and Harare, Zimbabwe (Clinicaltrials.gov NCT02732730). The study was initiated in October 2016 ahead of national PrEP guidelines and services for AGYW as a vanguard study, and required extensive community engagement and education to provide accurate information about PrEP identify and address PrEP-related myths and concerns, and promote PrEP acceptance and adoption in AGYW in the study communities in South Africa and Zimbabwe (3).

Stakeholder engagement and outreach were considered to be particularly essential given the target population was AGYW who frequently experience barriers in access to routine health care services because of inconvenient opening hours, challenges with transport to clinics, concerns about confidentiality, and judgmental attitudes about sexuality from health care providers (13, 14). The HPTN 082 community engagement teams provide lessons learned from recruiting AGYW into an open label oral PrEP study,reflecting on the experiences and strategies employed during the planning, readiness, and implementation phases including stakeholder engagement, PrEP education, and the creation of youth-friendly services. The insights provided aim to guide future PrEP introduction and service delivery efforts, ensuring high uptake and adherence among African AGYW.

### Good Participatory Practices (GPP) Framework

The UNAIDS/AVAC Good Participatory Guidelines provide a framework for effective and systematic engagement with stakeholders in the design and conduct of clinical trials throughout the trial life cycle (15). These guidelines were developed in part in response to the premature closure of two oral PrEP trials. Insufficient stakeholder engagement was viewed as one of the critical reasons for these closures, which were subsequently associated with delays in access to PrEP for vulnerable populations(15). Given this history, the HPTN 082 team was aware of the risks that inadequate community engagement could have in fuelling negative beliefs and fostering community mistrust, with subsequent impacts on trial conduct and outcomes. Furthermore, these negative impacts could extend beyond the trial and undermine the future uptake of PrEP in national programmes (16–18). Additional considerations related to the sensitivity of inclusion of sexually active adolescents and youth in the study given the barriers that adolescents face in accessing quality reproductive health care in many countries in sub-Saharan Africa.

Using the GPP framework, this manuscript reflects on lessons for stakeholder engagement and trial conduct from HPTN 082 and highlights those that are relevant for PrEP introduction and service delivery that leads to high PrEP uptake and coverage for AGYW more generally. The GPP guidelines are not prescriptive, instead, they provide research teams with a flexible framework to inform decision-making when engaging with stakeholders. While the lessons learned relate to oral PrEP, many of these have relevance for the Dapivirine Vaginal Ring (DVR) and long-acting injectable cabotegravir (CAB) as they become increasingly available in sub-Saharan Africa (19–21).

## Methods

### Study design

The HIV Prevention Trials Network (HPTN) 082 was an open-label study of PrEP uptake and adherence conducted between October 2016 and October 2018 among 16- to 25-year-old women without HIV in Cape Town and Johannesburg, South Africa, and Harare, Zimbabwe, as previously reported (11). Of the 451 AGYW enrolled, 427 (95%) initiated oral PrEP, with 412 starting at enrolment and an additional 15 during follow-up. Among these participants, 212 were randomized to the standard adherence arm, while 215 were allocated to the enhanced adherence arm which included adherence counseling based on drug level feedback provided at months 1 and 3. Thirty-one percent had detectable tenofovir diphosphate levels at month 12, which did not vary significantly between the standard and enhanced adherence arms. Overall, PrEP uptake was high but adherence and persistence werelow over 12 months (11).

### Study setting.

HPTN 082 was conducted at three clinical research sites (CRS), including Emavundleni in Cape Town, Wits RHI Ward 21 in Johannesburg, South Africa, and Spilhaus in Harare, Zimbabwe (see Table 2). Established in 2006, the Emavundleni CRS, part of the Desmond Tutu Health Foundation (DTHF), is in New Crossroads Township in Cape Town, South Africa. The Emavundleni team already had experience working with AGYW in other studies, and investigators had previous experience with PrEP studies, but for the community team, this was their first encounter with PrEP. The Wits RHI Ward 21 CRS was established in 2016 and is based in Hillbrow in inner-city Johannesburg. Wits RHI has had a presence in the community since 1994. The community engagement team already had experience working with AGYW but was less familiar with PrEP despite institutional experience conducting PrEP trials. The Spilhaus CRS housed at the Zimbabwe National Family Planning Council Clinic at Harare Central Hospital, one of the four largest referral hospitals in Zimbabwe, was established in 1994. HPTN 082 provided the team with the first experience of working with adolescents, although the site had previous PrEP trial experience.

### Data sources and analysis

This paper is written from the perspective of community engagement team members working in the field across the three HPTN 082 sites. It represents the accumulated experience, reflections, and discussions of the authors over the life of the HPTN 082 trial during the period between 2016 and 2020. Most of the authors were involved in all aspects of the HPTN 082 trial implementation and have first-hand experience in implementing GPP within trials.

At a meeting held in September 2018 before the final study closeout, the community teams from each of the three sites met specifically to reflect on the lessons learned from community engagement in HPTN 082 and their relevance for future programmes. Each site prepared a presentation highlighting their stakeholder engagement activities, organized into three stages preparedness, recruitment, and retention. Community engagement staff then collectively reviewed the challenges experienced during each stage. The reflections from this meeting created the basis for this manuscript.

Subsequently, the authors supplemented this initial workshop with insights gleaned from the review of meeting reports and minutes conducted from trial inception to close out (see Table 3). These meetings included four in-person meetings before the start (May 2016), during (December 2016, October 2017), and towards the end of the study (September 2018). The community team also reviewed the minutes of protocol team calls throughout the study as well as monthly site-level community engagement reports, work plans, training materials, and Community Advisory Board (CAB) meeting minutes.

The lead author (MM) also held recorded virtual reflection sessions with each community engagement team in preparation for this paper. During these reflection sessions, participants were asked to share lessons they learned in recruiting and retaining AGYW, promoting oral PrEP uptake among AGYW, and engaging with various stakeholders (CABs, parents, Youth community organisations, etc).

The gathered data was organised and analysed following the GPP framework. Data was first organised into three phases of a clinical trial, namely: planning/readiness phase, implementation phase, and close out, analysis, and dissemination phase. Under each phase, implementation strategies and lessons learned were grouped into relevant key 16 Good Participatory practices. The Good Participatory practices include formative research activities; stakeholder advisory mechanisms; stakeholder engagement plan; stakeholder education plan; communications plan; issues management plan; site selection; protocol development; informed consent process; standard of HIV prevention; access to HIV care and treatment; non-HIV related care; policies on trial-related harms; trial accrual, follow up and exit; trial closure and results dissemination; and post-trial access to trial products or procedures. Table 1 shows all 16 practices that were implemented in the HPTN 082 trial highlighting practices that were embedded in the study protocol and ones led by community teams. This paper reviews the practices which were led by community teams.

### Lessons learned recruiting AGYW into an oral PrEP trial.

Before the enrolment phase of the HPTN 082 study, recruitment commenced with community teams across all sites conducting community dialogues and stakeholder consultation meetings. During these events, stakeholders representing leaders from civil society organizations, and health care providers focused on PrEP introduction, specifically addressing concerns, myths, effective education, and recruitment strategies. During this process, community teams across all sites learned important lessons about PrEP introduction, PrEP messaging, and the inclusion of AGYW in clinical trials. This section is comprised of the team’s reflections and lessons learned before and during the study.

### Planning and readiness phase

During the planning and readiness phase, the community teams engaged in several activities to develop study procedures, complete approvals processes, and overall initial phases of stakeholder outreach and engagement.

#### Early collaboration with stakeholders

Before the trial, the community teams across all sites engaged in various *formative research activities* such as stakeholder mapping, and consultations with advisory boards, that informed outreach activities (stakeholder engagement plan). This was essential for introducing the HIV prevention trial in a manner that was appropriate for AGYW and effective in navigating cultural and age-related considerations to achieve acceptability and uptake. During this phase, stakeholders played a significant role in community engagement at the three sites, developing appropriate messaging and advising on spaces friendly for AGYW (stakeholder advisory mechanisms). Specifically, the youth Community Advisory Boards (CABs) played a key role in helping sites identify appropriate stakeholders, including representatives from advocacy groups, Ward counsellors, churches, clinics, schools, and peer educators. Each site worked closely with its respective CABs to identify stakeholders, design youth-friendly services, and determine comprehensive and robust community engagement strategies. Since youth-friendly services were considered a critical element of study delivery, the youth CABs and local youth-focused organizations were instrumental in providing advice on youth-friendly clinic design and clinic flow. For instance, the Spilhaus team reached out to Pangea Zimbabwe AIDS Trust to learn how they structured their youth-friendly clinic. At Wits RHI, members of the youth CAB were asked to conduct a walkthrough of the clinic identify aspects that may act as barriers to access and share additional activities they would like to access while in the clinic.

Based on feedback from the various consultations, all sites made adaptations to clinic setup to make study clinics more welcoming to young people. The focus was on creating spaces for youth that were welcoming, e.g., clear signage and access, non-judgmental staff, and comprehensive, integrated services for young women including provision of contraception, condoms, and STI testing. Referral pathways for social and psychological services were also mapped out in advance. Given that participants were anticipated to still be in secondary school or tertiary study, spaces for studying as well as internet access were provided, along with refreshments, in the clinic waiting rooms.

#### PrEP education and awareness.

As indicated earlier, communities were naïve about oral PrEP; therefore, normalizing PrEP dialogue and narratives was essential to effective recruitment of AGYW aligned to the values of youth-friendly services. To achieve this, at study initiation study teams developed communications plans aimed at providing transparent and accurate communication with relevant stakeholders (communications plan; stakeholder education plan). Using the communications plans as a guide study teams provided community education through PrEP videos which were designed specifically for the study, and fact sheets provided by the National Department of Health which became available when PrEP was introduced to programmes, outreach activities, and community dialogues to raise PrEP awareness, knowledge, and acceptability. To explain PrEP effectiveness, community teams used familiar existing services, such as oral contraceptives, to frame education interventions. The Ward 21 CRS community team reflected that in the beginning, it was challenging to conduct recruitment in the absence of a national campaign such as She Conquers, a three-year national campaign rolled out across South Africa by the Department of Health, to tackle the multiple social and structural factors that influence the high rates of HIV infection among AGYW (22); after the She Conquers program was launched, it supported recruitment.

Community teams across all sites were concerned that stigma and misconceptions could interfere with recruitment, oral daily PrEP uptake, or trial conduct. These concerns challenged the teams to refine their PrEP messaging to ensure they addressed any potential myths, or incorrect or incomplete narratives in the community. One of the misconceptions from the community was that PrEP replaced condoms as a single comprehensive prevention option. This signalled to community engagement teams that it was necessary to highlight that daily oral PrEP could not prevent STIs or pregnancy and that other prevention measures should supplement PrEP, which only prevents HIV acquisition. Another example of refined messaging was the adaptation of the traditional ABC message (A-Abstain, B-Be faithful, C-Condomize) to include PrEP. A further example is the replacement of messaging about “risky behaviours and populations” perceived to be judgemental and stigmatising. HPTN 082 messaging evolved to provide a sex-positive theme focusing on celebrating individual agency in risk reduction. The ongoing consultation with relevant stakeholders helped study teams design effective communication strategies that helped create a supportive and conducive environment for trial initiation and implementation as recommended by GPP.

The initial PrEP guidelines and demonstration projects were conducted exclusively with sex workers. As a result, PrEP became inadvertently associated with sex work. This is a fallacy as PrEP was conceived as a population-wide prevention option and only associated with key populations because introduction was limited initially to some key populations due to operational challenges. Stakeholders were further concerned that research teams may be viewed as encouraging multiple and concurrent sexual partnerships without adequate prevention measures. Stakeholders also voiced out concerns associated stigma from emtricitabine/tenofovir for PrEP as part of the first-line regimen for HIV treatment. With PrEP introduction there was no messaging sensitising users that anti-retroviral drugs were used for both PrEP and Antiretroviral Therapy (ART). This resulted in potential stigma from conflating PrEP-use withH IV infection. As a result, community engagement teams in HPTN 082 sensitised trial stakeholders to the use of emtricitabine/tenofovir, as used i both for ART with other antiretrovirals and used for PrEP, and reinforced that this does not reference HIV status.

The HPTN study team provided additional support to all community teams by organizing a recruitment workshop prior to site activation (Johannesburg, May 2016). The workshop goal was to further strengthen recruitment messages and adherence materials to support protocol implementation, especially considering the challenges identified above. An important aspect of this experiential workshop was the focus on values clarification as well as practicing recruitment “pitches” to make it easier for community teams to have conversations about PrEP in the field.

### Implementation phase

This phase of the clinical trial includes the recruitment, screening, enrolment, follow-up, and exit of trial participants (23). Stakeholder involvement remains critical during this phase as it helps study teams identify and mitigate trial-related stigma, misconceptions, or miscommunication (23).

#### Peers as sources of information, support, and referral

Peer referral was an important source of recruitment across all three sites(11). Screened study participants were encouraged to reach out to their peers and invite them to screen for study eligibility. To strengthen PrEP education, the Wits RHI team developed a short video titled “Get PrEPared: What African Women Need to Know!” which was shared with participants who then forwarded it to their peers.

Participants who managed to bring three new participants to the study for screening were given PrEP branded t-shirts regardless of screening outcome. Community teams highlighted that this strategy encouraged participants to contribute to recruitment and to create PrEP awareness among friends and family members. The teams also believed that this strategy destigmatised PrEP and made it look acceptable for young women who wanted to prevent HIV acquisition. Community educators encouraged participants to bring partners, friends, or family members who were unaware of PrEP and wanted to learn more about it to the research sites. This strategy ensured social support of the participant, which in turn, community teams believed, supported adherence to the product requirements and retention. To encourage ongoing participation in the study, participants were rewarded with Institutional Review Board approved merchandise such as lip balm, water bottles, branded t-shirts, and sling bags as they achieved study milestones.

#### Peer support-based adherence clubs

Protocol-defined standardized adherence support activities were developed from the activation of the study aimed at facilitating adherence and retention of trial participants [6]. Adherence clubs were designed to promote peer learning and support by providing participants with a regular platform to share their experiences and receive advice. During the sessions, study staff were present to facilitate discussions and address any concerns or questions about PrEP. While the clubs’ focus was to assist with PrEP adherence, they worked well as a strategy for supporting AGYW in participating in clinical research.

The Emavundleni team also used the clubs to strengthen partnerships with other community stakeholders as sessions were held once a month at a school opposite the trial site. The team collaborated with Zimele, a Desmond Tutu Health Foundation comprehensive HIV prevention program for adolescents (24). The adherence club lasted an hour, followed by the Zimele program for those who had signed up. The overarching lesson learned by community teams was that clubs might not work for everyone and busy adolescents in school may not have time.

#### Key gatekeepers

Research teams started appreciating the important role played by parents as gatekeepers, after the trial started (stakeholder advisory mechanisms). The teams learned that early and ongoing parental involvement addressed research team concerns about getting buy-in from parents and guardians to give parental or legal guardian consent for young women under age 18 to participate in the study. They also learned that even those who could give consent were still influenced by parents. To address possible undue influence that would counter the objectives of the trial, the community teams held meetings with parents, organized through school governing bodies, to educate them about PrEP and to outline minors’ involvement in the study. The intentional engagement of parents highlights the GPP assertion that study team’s constructive engagement with stakeholders “deepens understanding of local context”.

Other parental concerns addressed by community teams were about potential disturbance to school attendance. They addressed this by scheduling study visits outside of school hours. At the Emavundleni site, some parents worried that the autonomy created by reimbursing adolescent participants would dilute parental authority. Furthermore, Zimbabwean parents raised concerns about long-term fertility prospects after prolonged use of oral contraception. Community teams addressed parents’ concerns through health education and dialogue.

In addition, some parents raised concerns related to post-trial access to PrEP (post-trial access to trial products or services). For study teams the transition to local services was important and flagged early on. Engaging stakeholders and participants on trial closure and results dissemination was key for study teams as they understood that it is essential for building trust and lays a positive foundation for future research (15). At the end of the HPTN 082 study, participants from Emavundleni and Ward 21 were referred to a PrEP demonstration project called POWER. At the Emavundleni clinical research site, they were also referred to other studies, and if they did not meet the eligibility criteria of that study, they would be referred to the nearest health facility. The only challenge the Emavundleni site had with participants was that some participants still wanted to continue getting contraceptives and the standard of care they were getting during the study.

#### PrEP delivery offered through adolescent and youth-friendly services.

As part of offering adolescent –and youth-friendly services (AYFS) and ensuring participants experienced clinic visits as beneficial, all sites focused on improving efficiency and clinic flow with such strategies as pre-booking, batching participants, and having adequate clinical staff coverage. The sites implemented simple system changes that showed significant improvement in clinic flow and shortened waiting times. In addition to more effective flow, participants were engaged in activities meaningful to their life progress such as workshops to develop their resumes.

Research sites implemented a variety of strategies to maintain communication pathways between participants and study staff. For instance, at Emavundleni, the participant’s initial recruiter remained the staff person focused on retention, strengthening trust, and improving communication. Research teams developed flexible scheduling regimes that allowed for early morning/evening/weekend clinic hours or priority days for clinic visits. Across all sites amenities such as childcare and commodities were made available to support study participants with children so they could attend their appointments without the burden of finding alternative childcare. Amenities such as computers, free Wi-Fi, mini-library, television, coffee/tea, and meals were provided to improve the sensory comforts associated with prioritized care.

#### Retention support for AGYWparticipants

Community Engagement teams emphasised that a focus on retention should commence as early as pre-screening and field recruitment. The first contact participants have with a study often influences their expectations throughout. Retention outreach teams were socialized and trained to create good rapport from the first encounter, which was continued throughout the study experience. Paying attention to retention risks upfront also mitigates possible losses later. This was validated by a locator form to capture participant’s location, contact details, and alternative contacts. Adequately captured locator information increase contact effectiveness and participant retention so, at every site visit, this form was revalidated frequently together with the documentation of retention or adherence concerns.

#### Participants mobility

One factor that affected retention was participants taking unplanned travel or making alternative living arrangements. To overcome this problem, Spilhaus conducted rural outreach to pick up participants who had moved to rural areas. After the clinic visit, participants would be reimbursed for transport to return home. A related factor was high mobility among students, largely experienced by the Ward 21 and Emavundleni teams. Reasons included students graduating from universities in these cities and moving either back to their homes, to a new location for work, or to a town where their parents had moved; or they were too busy with school. Some study sites endeavoured to transport out-of-town participants. In Ward 21 and Spilhaus, the community teams acknowledged that priming participants about 12-month commitments and probing future plans can, to a degree, avoid enrolling those who may be at high risk for loss-to-follow-up or premature exit.

#### Engaging with AGYW versus engaging adults older than 25

Teams learned that the level of participant engagement required for adolescents is different than for adults which in prior clinical research studies had high retention with minimal engagement. With adolescents, engagement was more deliberate as they tend to lose interest easily(11). Community teams learned that young people could feel fatigued by the ongoing narrative about their risk for HIV and prefer to speak about their wide range of interests as well. Quarterly retention events incorporated1 entertainment (e.g., dance, movie day, aerobics sessions), and 2 skills training (e.g., writing a curriculum vitae, self-defence classes, makeup tutorials). Participants were encouraged to bring their friends, relatives, and partners. The events served as an effective way to meet between the actual study visits, and messages about their contribution and why their role required study completion were emphasized. Participants also had opportunities to build networks among themselves to share experiences, which was socially rewarding.

## Discussion

The HPTN 082 trial was one of the early oral PrEP studiesin Southern Africa and which presented the HPTN 082 community engagement teams with many initial challenges associated with engaging AGYW who had limited prior experience of taking an oral daily pill for HIV prevention. The major concerns AGYW expressed were about side effects and the burden of daily pill-taking (25), and stigma linked to using medications associated with HIV treatment, as others could mistakenly assume that they were living with HIV (26). However, HPTN 082 and past studies (26–29)idemonstrated that AGYW can accept and adopt new HIV prevention methods such as PrEP when there is a focus on community involvement, education, and appropriate messaging.

This paper has highlighted nine key community engagement lessons when introducing new PrEP products, including the importance of early engagement with stakeholders; the necessity of PrEP discussion, education, and awareness; the pivotal role of peers as sources of information, support, and referrals; the facilitation of open dialogues through peer support-based adherence clubs; the influence of key gatekeepers on AGYW; the importance of delivering PrEP through youth-friendly services; the need for retention support for AGYW participants; the anticipation of participants’ mobility requirements; and the distinct nature of engaging AGYW as trial participants compared to adults.

These lessons were derived from the dedicated time and effort invested by the HPTN 082 community teams in engaging with AGYW and various stakeholders, including peers, partners, and parents. This engagement with AGYW influencers resulted in positive outcomes, as they contributed to recruitment and retention strategies and supported PrEP uptake and adherence. Conversely, inadequate involvement of stakeholders can hinder PrEP adoption, as demonstrated in other research studies (25). This underscores the importance of future PrEP studies or programs prioritizing engagement strategies that actively involve stakeholders, as AGYW relies on their support to navigate discussions concerning PrEP usage, adherence, and acceptance.

The GPP framework played an important role in shaping effective community engagement strategies in the context of introducing new HIV prevention methods such as PrEP. GPP emphasises the importance of early collaboration with stakeholders, transparent and accurate communication, stakeholder education, and the involvement of key gatekeepers such as parents and community leaders. By implementing GPP, research teams can navigate cultural nuances, address myths and misconceptions, and foster trust within communities, especially among AGYW. As the HIV prevention landscape continues to evolve, GPP remains a valuable framework for guiding community engagement efforts and ensuring the successful implementation of PrEP and other HIV prevention interventions.

One notable limitation of this study is the risk of recall bias by the community educators who retrospectively considered lessons in engaging AGYW and stakeholders. The effectiveness of individual strategies were not assessed, but the overall consensus was the need for intentional design in community engagement strategies and activities prior to introducing new products or services. These retrospective observations can inform community engagement strategies implemented for a PrEP demonstration project early in PrEP rollout in South Africa and Zimbabwe but may not be feasible or generalizable to programmatic scale-up.

## Conclusion

The HIV prevention landscape continues to evolve, and the need to effectively engage adolescents in HIV prevention options remains a high priority. The Good Participatory practices shared in this paper provide insights on how to think about and develop effective community engagement strategies when introducing a new HIV prevention method or technology such as PrEP. There is no one-size-fits-all approach to engaging AGYW and implementing PrEP; a local-level understanding is critically important for successful implementation. The GPP guidelines are recommended to help research teams prepare, recruit, and retain this population given their dynamic daily needs. Lessons shared in this paper can also inform policy and programming for oral PrEP as well as the expanding options of ARV-based PrEP both in terms of drugs being evaluated and vehicle of delivery.

## Figures and Tables

**Table 1:** HPTN 082’s Good Participatory Practice Implementation Model (adapted from the Wits RHI GPP Implementation Model(23))

	GPP Topic Areas	Tools and Participatory Strategies*	Stakeholders Engaged and Output*	GPP Principles and Value-Added Outcome
Planning/Readiness Phase	Formative Research Activities	GPP included in grant proposals/study budgets	GPP staff hired, resources allocated, training and activities budgeted upfront into clinical research/implementation project.	Research/project held accountable for optimal engagement when resources are sufficient.
		Stakeholder mapping and analysis	Stakeholders identified and prioritised, which informs engagement platforms and strategies.	Research team’s socio-cultural competency strengthened contributing to mutual understanding.
		Informal focus groups	Research design, data collection, implementation informed and reviewed by Community Reps (Key Pop, NGOs, faith-based, police students)	
	Stakeholder Advisory Mechanisms	Youth CAB, Prevention CAB, Local youth-focused organisations	Research design, data collection, implementation informed and reviewed by key stakeholders.	Ongoing stakeholder engagement contributes to scientific and ethical integrity, accountability, transparency, and mutual understanding. Enabling environment improves protocol/project design, implementation, and oversight.
		Structured consultations, events, and workshop		
	Stakeholder Engagement and Stakeholder Education Plan	Structured template plans, Partner MOUs, Research literacy, and issues-based training	Stakeholders receive understandable (and translated) materials and resources; Research literacy capacity strengthened.	
	Communications Plan	Communications plans	Strategic communications plans outlining which stakeholders will be engaged, when, and how.	Equipped stakeholders to proactively manage communications and issues transparently and accountability
	Issues Management Plan	SOPs and systems to prevent, manage and escalate issues	As appropriate, sponsor, staff, participants, CABs notified; guidance sought if issues arise.	
	Site Selection	GPP included in site selection process and REC application	Research site assessed on track record and (if new site) commitment to GPP	Stakeholder engagement mechanisms developed and or assessed.
	Protocol Development	GPP incorporated into study protocol and documents	Research team formally committed to upholding GPP principles and undertaking GPP	Stakeholders maintain autonomy; process promotes respect. Consultation reports aim to provide transparency and mutual understanding (even if agreement not always reached).
Implementation phase (trial accrual, follow up and exit)	Informed Consent Process (icp)	Informed Consent SOP; ICP assessment of understanding	Staff trained on ICP Trained, CABs and advocates review and provide input/feedback to ensure ICP is age and culturally appropriate for the intended community and participants.	Locally acceptable, understandable, and transparent informed consent procedures and materials developed.
		ICP Stakeholder review		
	Trial Accrual Follow Up, and Exit	Recruitment/Retention SOPs	Range of retention events; clubs/ peer support groups for participants; parents engagement events; community dialogues utilised as appropriate.	Multiple engagement mechanisms ensure suitable interventions respond to the needs of participants and families.
		Community Engagement Work Plans	Libraries, free Wi-Fi, baby cots, children’s area, refreshments improve clinic environment.	Informed socioculturally acceptable ways of recruiting, retaining, and exiting participants strategies.
		Adolescent and Youth-friendly clinics	Flexible clinic hours based on participant cohorts’ needs improve accessibility.	
	Standard of HIV Prevention	Prevention packages align with national guidelines, including oral PrEP where applicable	Participants receive comprehensive protocol specified HIV prevention package.	Up to date prevention packages maintain respect and accountability of research teams to participants.
	Access to HIV Care and Treatment	Counselling; robust referrals; peer navigators; linked database	Participants who seroconvert are transferred to care and treatment programme of choice.	Strong linkage to HIV and non-HIV related care provided to participants as needed fosters respect, greater trust, and accountability of research teams to participants.
	Non-HIV Related Care	Study protocols and / national guidelines	STI testing and treatment, contraception, psycho-social support services.	
	Policies on Trial-Related Harms	Counselling; Referral SOP; CBO partnerships; Resource guide	Support services / resources provided directly and discreetly available to participants.	Staff offer holistic care and respect participant’s right to use or refuse referral, within legal reporting obligations.
		Social harms reporting: safety planning as needed	Participants counselled, offered referrals, and followed up with support.	
Close out analysis & dissemination phase	Trial Closure and Results Dissemination	Outcome scenario planning, dialogue workshops	Civil society, researchers, and communicators prepared for possible outcomes; Q&As and messages developed.	Dissemination strategies informed and stakeholders’ expectations managed through transparent engagement,
		Dissemination strategy and activities	Publications, media, events, and videos used to disseminate to range of stakeholders.	Research outcome disseminated widely using multiple platforms - ensuring scientific integrity, transparency and contribution to the evidence base.
		Research uptake strategy; policy briefs, technical working groups	Policy, guidelines, public health programmes and training tools informed and developed.	
	Post-trial Access to Trial Products or Procedures	Post-trial access in: protocol; open-label extensions; or negotiated with partners	Post-trial access for participants is protocol (and often donor) dependent and time bound.	Aim maximise benefits post-trial to participants maintains accountability.

***Key:*** Shaded boxes in columns 2 and 3 are activities that were embedded in the HPTN 082 protocol, and the unshaded boxes were led by community teams.

**Table 2.** Study setting and community engagement team composition and experience.

CRS, location	Study setting characteristics	Composition of community engagement teams	Previous experience with AGYW	Previous PrEP experience
Emavundleni, South Africa Location: New Crossroads Township, Cape Town	Poor housing (27) Crime and violence (27) High levels of poverty High unemployment rate High HIV incidence (28)	CLO n=1, Community educators n=7, 17-plus years collective experience in community engagement in research.	Yes	Yes
Wits RHI Ward 21, South Africa Location: Hillbrow, Johannesburg	Densely populated (29) Highly mobile population (30) Highly migrant population (29) Poor housing (30) Student population (30) High unemployment rate High prevalence of HIV/TB Crime and violence	Community engagement manager n=1 CLO n=1 Community educators =2, 17-plus years collective experience in community engagement in research.	Yes	Yes
Spilhaus, Zimbabwe Location: Harare Hospital, Harare	Densely populated (31) High unemployment rate (32) High rates of intimate partner violence (32) Child-headed households (31) High HIV prevalence (32) Low socioeconomic status Drug abuse	CLO n=1, Community educators n=3, 10-plus years collective experience in community engagement in research.	No	Yes

* CLO: community liaison officer

**Table 3.** Data sources and topics

Meeting type, location, and date	Good participatory practices addressed	Participants and size	Data source and collection
Recruitment messaging workshop, Johannesburg, May 2016	Stakeholder engagement and education Strategies to message PrEP Strategies to overcome barriers (engage with all stakeholders, prioritize community education, address all misconceptions/myths/rumors timely, male involvement). Trial conduct Trial staff values clarification exercises Community engagement lessons learned from previous studies. Barriers to recruitment include myths and misconceptions, parental consent, staff attitudes, clinic waiting times.	HPTN 082 protocol leadership, community teams from all 3 sites n= Unknown	Meeting notes
Protocol team call 02 February 2017	Stakeholder engagement and education Oral contraceptives used as an example to explain PrEP effectiveness and time to protection. Condom use in the context of oral PrEP use; discussions about condom use particularly when first starting PrEP	Protocol team including protocol chairs, study teams including community educators, and representatives from HPTN coordinating, lab and data centre n= 27	Meeting minutes
Protocol team call 04 May 2017	Stakeholder engagement and education Community attitudes identified as a barrier to adherence because of stigma associated with antiretroviral pills and concerns about disclosure of sexual activity Context of limited community information about PrEP in country identified as important barrier Recommendations to share positive information about PrEP through notice boards and other community education venues Trial conduct Parental consent requirements and disclosure of trial participation to parents identified as a barrier to recruitment and retention Potential for inadvertent disclosure when traveling with pills identified as a barrier to adherence	Protocol team n=19	
Protocol team call 07 September 2017	Stakeholder engagement and education Frequent myths and rumors about PrEP research participation and blood sample collection identified Concerns and associations between PrEP use and having multiple sexual partners identified.	Protocol team n=26	
Protocol team meeting at HPTN Regional Meeting, Johannesburg 17 October 2017,	Stakeholder engagement and education Research literacy involves translating scientific jargon into everyday language and images that people can relate to. Important to use scenarios and narratives as part of community education Recognize need to put community education at the heart of every activity and forge strategic partnerships with key stakeholders. Parents and other adults are key gate keepers for AGYW use; materials need to speak to adults as well as AGYW concerns and facilitate or support disclosure discussions Use holidays & National Health Calendars to locate the study within broader sexual and reproductive health and HIV prevention issues. Trial conduct AGYW have expressed concerns about ongoing access to PrEP post-trial. Sites need to develop plans regarding post-study access.	Protocol team n not recorded	Meeting report
Protocol team call 02 November 2017	Trial conduct Focus on plans to manage retention and adherence during upcoming holidays. Plans for “PrEP for Christmas” parties or events to make PrEP & study participation fun and engaging for AGYW.	Protocol team n=26	Meeting minutes
Protocol team call December 2017	Trial conduct Retention and adherence plan across all sites.	Protocol team n=19	
Protocol team call 11 January 2018	Trial conduct All sites provided updates on steps they took to maintain high retention over 2017 December holidays.	Protocol team n=25	
Protocol team call 5 April 2018	Stakeholder engagement and education Discussion regarding development of site-specific short films to communicate AGYW lived experiences of taking PrEP Status of video vignettes (ethics approval, filming, editing etc).	Protocol team n=27	
Protocol team call 3 May 2018	Stakeholder engagement and education Preparing for dissemination of results. Discussion about rough cuts for short films from two sites Trial conduct Focus on retention for the final visits, Ensuring post-trial access to PrEP for young women who are interested	Protocol team n=21	
Community Engagement team Manuscript writing meeting, Cape Town September 2018	Stakeholder engagement and education Lessons learned regarding stakeholder and community mapping, community outreach drives and the development of partnerships and collaborations with stakeholders. Community dialogues as a strategy for stakeholders’ consultation and engagement Challenges in introducing a new HIV prevention method to people and communities with little or no knowledge about PrEP required a lot of explaining, patience and understanding. Value of youth friendly, sex positive multimedia recruitment and educational materials; PrEP film and the PrEP material helped in explaining and educating the stakeholders. Trial conduct Best practices for recruitment identified across all 3 sites including peer to peer recruitment. Barriers to recruitment at schools and focus on recruitment at tertiary institutions	Community teams from all 3 sites n=8	Meeting minutes
Protocol team call 1 November 2018	Stakeholder engagement and education Planning results dissemination.	Protocol team. n=21	

## Data Availability

All audiotapes and transcripts of interviews with community teams, meeting minutes, and workshop summaries are stored at Wits RHI, South Africa. They are available from the study corresponding author on reasonable request.

## Abbreviations

AGYW: Adolescent Girls and Young Women
ART: Antiretroviral Therapy
AYFS: Adolescent-and-youth-friendly services
CAB: Community Advisory Board
GPP: Good Participatory Practice
HIV: Human Immunodeficiency Virus
HPTN: HIV Prevention Trials Network
IRB: Institutional Review Board
NDOH: National Department of Health
PrEP: Pre-Exposure Prophylaxis
TB: Tuberculosis
WHO: World Health Organization
